# The optimal mid-upper-arm circumference cutoffs to screen severe acute malnutrition in Vietnamese children

**DOI:** 10.3934/publichealth.2020016

**Published:** 2020-03-23

**Authors:** Tran Thi Hai, Saptawati Bardosono, Luh Ade Ari Wiradnyani, Le Thi Hop, Hoang T. Duc Ngan, Huynh Nam Phuong

**Affiliations:** 1 Southeast Asian Ministers of Education Organization Regional Center for Food and Nutrition (SEAMEO RECFON)/Pusat Kajian Gizi Regional (PKGR), Jakarta, Indonesia; 2 Department of Nutrition, Faculty of Medicine, Universitas Indonesia, Dr. Cipto Mangunkusumo General Hospital, Jakarta, Indonesia; 3 Department of Nutrition, Faculty of Environmental and Occupational Health, Hanoi University of Public Health, 1A Duc Thang Road, Duc Thang Ward, North Tu Liem District, Hanoi, Vietnam; 4 Vietnam Nutrition Association, Hanoi, Vietnam; 5 National Institute of Nutrition, Hanoi, Vietnam

**Keywords:** optimal MUAC cutoff, severe acute malnutrition children, mid-upper-arm circumference, wasting children, MUAC

## Abstract

Severe acute malnutrition (SAM) remains a main cause of mortality among children under five years old. Vietnam needs further study to establish the optimal mid-upper-arm circumference (MUAC) cutoff for improving the accuracy of the MUAC indicator in screening SAM children aged 6–59 months. A survey was conducted at all 16 subdistricts across four provinces in Northern Midlands and mountainous areas. The data of 4,764 children showed that an optimal MUAC cutoff of 13.5 cm would allow the inclusion of 65% of children with weight-for-height z-scores (WHZs) below −3SD. A combination of MUAC and WHZ may achieve a higher impact on therapeutic feeding programs for SAM children. The MUAC cutoff of 13.5 cm (65% sensitivity and 72% specificity) should be used as the cutoff for improving and/or preventing SAM status among children under 5 in the Midlands and mountainous areas in Vietnam.

## 1. Introduction

Severe acute malnutrition (SAM) remains a main cause of mortality among children under five years old. SAM (weight-for-height/weight-for-length z-score [WHZ] less than −2SD) also affected nearly 52 million children under five [1]. SAM children have an approximately 10 times higher risk of mortality than their well-nourished peers [2]. It is estimated that the identification and management of SAM could prevent over 400,000 child death per year [3].

Since 2013, the WHO guidelines have stipulated the use of MUAC at the community level to screen for SAM, while healthcare workers in primary healthcare facilities and hospitals should assess either MUAC or WHZ status and also examine the bilateral edema in infants and children aged 6–59 months. Children with medical complications and SAM are treated as inpatients in facilities and hospitals, whereas, children without medical complications and SAM are treated at home, following a community-based program for the management of acute malnutrition (CMAM) [4]. Since early detection using MUAC and WHZ criteria was implemented, 90–95% of affected children can be treated in the community [5]. Ready-to-use therapeutic foods have been also supported in CMAM programs [6]. In 2014, Vietnam joined five Asian countries that also adopted CMAM programs to provide care for children with acute malnutrition [7].

A MUAC cutoff less than 11.5 cm and/or a WHZ less than −3.0 is currently recommended to detect SAM in all children aged 6–59 months [4]. A cutoff between 11.5 cm and 12.5 cm is recommended to diagnose moderate acute malnutrition (MAM) children. However, several studies found that MUAC and WHZ indicators could detect different acute malnutrition children [8],[9]. The findings from several studies showed that the MUAC indicator's accuracy could be improved by different cutoffs [10],[11]. Screening acute malnutrition at the community level requires validating the MUAC cutoff in a Vietnamese setting, for which there is a paucity of data.

## 2. Methods

### 2.1. Data collection

A community-based cross-sectional survey was conducted in 16 subdistricts of four provinces in the Northern Midlands and mountainous area in Vietnam. Children aged 6–59 months were screened for acute malnutrition using weight, MUAC, height (or length for children aged less than 24 months), and the presence of bilateral edema. The survey included all children aged 6–59 months with no medical complications. The recumbent lengths of the children aged less than 24 months and the height of the children aged 24–59 months were measured using a microtoise (UNICEF), and the measurements were recorded to the nearest 0.1 cm. The children's weight was calculated with a SECA electronic scale following the international recommendations and also recorded to the nearest 100 g. The MUAC measurements were made using a nonstretch tape measure (SO145620, MUAC, Child 11.5 Red/PAC-50) provided by the National Institute of Nutrition in Vietnam. To determine the children's ages (in months), their birth dates were either extracted from official documents (e.g., refugee registers), or mothers/caregivers were asked. Training was conducted for all the measurers (NIN health workers). Calibration of the measurement tool was done before the data collection. All data were recorded for each child.

### 2.2. Statistical analysis

The WHZs (WHO 2006 growth standards) and the other anthropometric indices were calculated using the WHO Anthro for PC software [12]. The data were initially cleaned by deleting the outliers using set flag limits. Records with any of the following criteria were also excluded:

1. Age less than 6 months or more than 59 months
2. WHZ or HAZ or WAZ or MUAC was not available

All analyses were performed in SPSS 20.0 for Windows (SPSS Inc., Chicago, USA). The sensitivity, specificity, and positive predictive values of the MUAC were determined using WHZs as the gold standard. Receiver-operator characteristic (ROC) curves were constructed to present the relationship between the MUAC and WHZ for different cutoffs. The Kappa statistic (k) was calculated, and the association test was considered excellent at k > 75%, good at 75% ≥ k ≥ 40%, or marginal at k < 40%.

The data analysis was carried out at the SEAMEO Regional Center for Community Nutrition in Jakarta, Indonesia.

### 2.3. Ethical considerations

Before agreeing to participate, the children's mothers/caregivers were informed about the purpose of the study and the name of the research institution. As research participants have the right to skip any steps of the survey or to refuse to participate without penalty if they wish, no negative consequences resulted to those who decided not to participate. The cooperation of all participants was voluntary. The collected data will be used for study purposes only. The study protocol was approved by the Ethical Committee of the National Institute of Nutrition in Hanoi, Vietnam (number 844/VDD-QLKH on November 12, 2015).

## 3. Results

A sample of 5,098 children was included. After the removal of 182 outliers, 78 subjects aged more than 59 or less than 6 months and 74 subjects missing MUAC values, 4,764 individuals (more than 93%) contributed to the further analysis.

### 3.1. Comparison of MUAC with WHZ

Of the total 4,764 children aged 6–59 months, 50.3% were male. The median age was 32.6 months (19.4, 45.2). The prevalence of 24–59 month-age children was two times higher than that of children aged 6–23 months, which was 34.1%. The mean MUAC was 14.0 ± 1.1 cm and ranged from 10.0 cm to 21.0 cm. The prevalence of SAM as indicated by a MUAC less than 11.5 cm was only 1.3%. In comparison, the prevalence of SAM as indicated by WHZ was two times higher, at 2.3%. However, the prevalence of MAM as indicated by a WHZ between -3SD and -2SD, was 6.1%, which was smaller than that of MAM as determined by a MUAC between 11.5 cm and 12.5 cm (8.0%). The current MUAC cutoffs for diagnosing global acute malnutrition (GAM; a MUAC less than 12.5 cm), MAM (a MUAC between 11.5 cm and 12.5 cm), and SAM (a MUAC less than 11.5 cm) compared poorly to the same categories as defined by WHZs, with Kappas of 16.4%, 11.8%, and 5.5%, respectively.

The sensitivity, specificity, and positive predictive values of each MUAC cutoff point compared to the WHZ were shown in Table 1, using 2 × 2 table calculations. For SAM, the MUAC cutoff value of 13.5 cm resulted in an absolute increase of 60% in sensitivity, a 27% decrease in specificity, and the highest sensitivity compared to the 11.5 cm cutoff.

### 3.2. ROC curves

**Figure 1.**
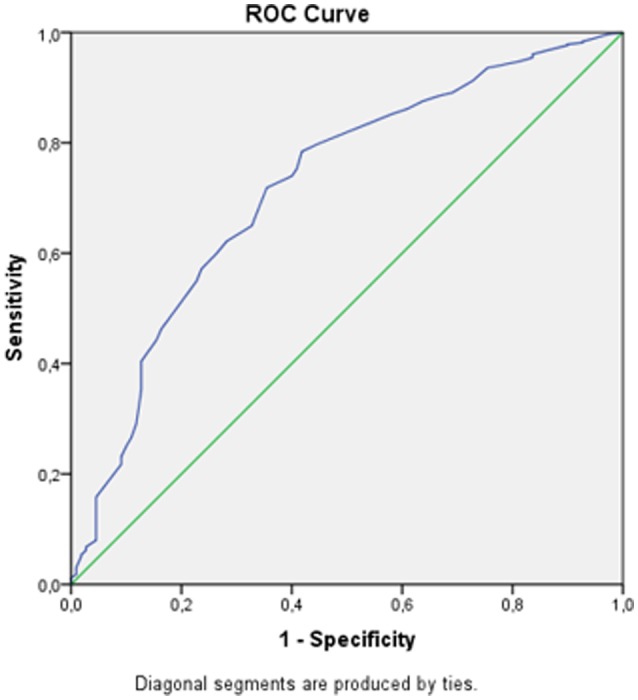
ROC curve of the MUAC score against a WHZ < −3SD.

To determine whether the power of the MUAC to predict GAM and SAM could be improved, ROC curves were drawn against a WHZ < −3S. The sensitivity, specificity, and positive predictive values of each MUAC cutoff were calculated and showed an optimal MUAC cutoff of 13.5 cm for detecting SAM. In Figure 1, an ROC curve was drawn using a nonparametric method in the SPSS software (AUC = 0.72, 95% CI: 0.67–0.77, p < 0.001). This curve and the corresponding area under the curve (AUC) show that the MUAC indicator as a proxy has the predictive ability to discriminate SAM children from normal children aged 6–59 months.

**Table 1.** Comparison of Different MUAC Cutoff Points and WHZs among Children Aged 6–59 Months.

WHZ < −3SD				WHZ < −2SD			
MUAC cutoff (cm)	Sensitivity	Specificity	Positive predictive value	MUAC cutoff (cm)	Sensitivity	Specificity	Positive predictive value
11.5	0.05	0.99	0.10				
				12.5	0.25	0.92	0.23
13.1	0.55	0.80	0.06	13.1	0.48	0.81	0.19
13.2	0.58	0.78	0.06	13.2	0.50	0.80	0.19
13.3	0.59	0.75	0.05	13.3	0.54	0.77	0.18
13.4	0.60	0.74	0.05	13.4	0.55	0.76	0.17
**13.5**	**0.65**	**0.72**	**0.05**	13.5	0.57	0.74	0.17

## 4. Discussion

In our data set, the MUAC cutoff of 13.5 cm resulted in an absolute increase of 60% in sensitivity, a 27% decrease in specificity, and the highest sensitivity in screening SAM compared to the 11.5 cm cutoff. Our findings are consistent with those reported. A previous study of 39 surveys conducted in a sample of children aged 6–59 months in 10 mostly African countries showed that a MUAC cutoff of 13.5 cm, which is the same as our optimal cutoff, was optimal for identifying SAM [10]. The results from the individual countries are also close to our findings. The optimal MUAC cutoffs for Cambodian and Indian children were 13.3 cm and 12.0 cm, respectively [11]. For the Indian setting, the best cutoff of 12.0 cm, which was found in hospital-based enrollment wherein only underweight children were included, was 1.5 cm less than our result. A change in the MUAC cutoff value is needed [13].

Conversely, the higher MUAC cutoff point for detecting acute malnutrition in admission to CMAM also had a lower specificity. Moreover, the data from several countries showed that the higher MUAC cutoff point should be used as a criterion for hospital discharge [14],[15]. Thus, the MUAC cutoff of 13.5 cm should be used as the cutoff for improving and/or preventing SAM outpatients. For SAM inpatients, the higher MUAC cutoff point should be used as a criterion for hospital discharge. A further study is needed to follow-up children under 5 having a MUAC ≤ 13.5 cm. After that, monitoring and discharge criteria for inpatients and outpatients should be implemented to revise the appropriate indicator threshold. That our study is only a survey could be a limitation.

However, identifying different subgroups of children at risk of death using the MUAC and WHZ was understandable because of the MUAC concepts. A study measuring MUAC and triceps skinfold thickness showed that MUAC was strongly related to fat mass in children but poorly related to fat-free mass or overall weight [16]. In contrast, the WHZ strongly negatively relates to body weight. In our study, skinfold thickness indicators were not assessed. Moreover, the MUAC indicator for diagnosing SAM based on MUAC relies on a single absolute cutoff point independent of age, height, and gender. However, the MUAC increased steadily as the children's height/length and weight increased. Thus, children were more likely to have fallen below the absolute MUAC cutoff point if they were younger. In our data set, children aged less than 24 months of age were at a higher risk of SAM and were identified by a MUAC less than 11.5 cm (r^2^ = 1.42, p < 0.001) (the data not shown). Overall data set between the WHZ and MUACz distribution was be shown in Appendix 1. A large data set from 47 countries measuring children aged 6–59 months also showed that MUAC increased with children's age and height [17]. A cohort study among children aged 12–59 months also showed that the MUAC indicator tended to identify significantly more younger children than those identified by the WHZ [18].

Countries having a higher proportion of shorter children have fewer children diagnosed with acute malnutrition by WHZ and thus more by MUAC alone. The positive association between longitudinal growth (stunting) and ponderal growth (wasting) would increase the proportion of children with low WHZs, while MUAC is not influenced. However, a large data set from 47 countries found a weak relationship between the prevalence of stunting and fewer incidences of GAM detected by WHZ and more by MUAC alone (r^2^ = 0.19, p < 0.01) [17]. Our results showed a high prevalence of simultaneous stunting and wasting, and the prevalence of SAM based on a WHZ less than −3SD was higher than that based on a MUAC less than 11.5 cm.

The sitting-to-standing height ratio (SSR) can affect the WHZ, whereas the MUAC is independent. Additionally, long-leggedness may be disadvantageous when we calculate the WHZ because the legs weigh less per unit length than the torso. Thus, in some children, a low WHZ is not as serious as a low MUAC in a nutritional assessment. Some studies found a relationship between different SSRs and different livelihood zones. The SSRs of a pastoralist population were lower than those of the settled population [19]. Besides, an observation study showed that populations from cold climates tend to have shorter limbs than those from warm climates. Moreover, the SSR also tended to be lower in populations from areas with higher mean temperatures [20]. That the target subjects of our study live in midland and mountainous areas may be the reason for the discrepancy between the two indicators in detecting acute malnutrition. However, in our data set, the effects of WHZ, MUAC, SSR, or even livelihood zones and climate on the clinical and physiological outcomes of children were not explained.

Although the assessors, who had public health or medical doctor backgrounds, received training, and the outliers were few, the lack of testing the measurers' precision and accuracy was a limitation [21]. A study with a larger sample size is recommended to establish an age-specific MUAC cutoff among the relationships of different factors such as stunting status, SSR, body composition, livelihood zones, and climate. Moreover, medical checking should be the gold standard for diagnosing SAM. However, the limited resources prevented us from conducting a perfect survey in the Northern Midlands and mountainous areas in Vietnam. A prospective study working on how different anthropometric indices predict disease-specific morbidity and mortality is also needed [21].

## 5. Conclusion/Recommendation

MUAC is considered a quicker, lighter, cheaper, and more robust screening tool at the community level than WHZ. Using a broader cutoff of 13.5 cm raises the sensitivity of the MUAC indicator from 5% (at a cutoff of 11.5 cm) to 65%. Hence, the combination of MUAC and WHZ may achieve a higher impact on the therapeutic feeding program for SAM children. The MUAC cutoff of 13.5 cm (65% sensitivity and 72% specificity) should be used as the cutoff for improving and/or to preventing SAM status among children under 5 in the midland and mountainous areas in Vietnam.
