# Supplementary material for: The optimal mid-upper-arm circumference cutoffs to screen severe acute malnutrition in Vietnamese children
Source: AIMS Public Health. 2020 Mar 23;7(1):188–96. doi: 10.3934/publichealth.2020016 (PMC7109525; doi:10.3934/publichealth.2020016)
Supplement: Supplementary file 1 [file publichealth-07-01-016-s001.pdf]

Public Health

AIMS Public Health, 7(1): 188–196.
DOI: 10.3934/publichealth.2020016
Received: 10 December 2019
Accepted: 17 March 2020
Published: 23 March 2020

http://www.aimspress.com/journal/aimsph

Research article

The optimal mid-upper-arm circumference cutoffs to screen severe acute malnutrition in Vietnamese children

Tran Thi Hai[1,2,3,*], Saptawati Bardosono[2], Luh Ade Ari Wiradnyani[1], Le Thi Hop[4], Hoang T. Duc Ngan[5] and Huynh Nam Phuong[5]

[1] Southeast Asian Ministers of Education Organization Regional Center for Food and Nutrition (SEAMEO RECFON)/Pusat Kajian Gizi Regional (PKGR), Jakarta, Indonesia
[2] Department of Nutrition, Faculty of Medicine, Universitas Indonesia, Dr. Cipto Mangunkusumo General Hospital, Jakarta, Indonesia
[3] Department of Nutrition, Faculty of Environmental and Occupational Health, Hanoi University of Public Health, 1A Duc Thang Road, Duc Thang Ward, North Tu Liem District, Hanoi, Vietnam
[4] Vietnam Nutrition Association, Hanoi, Vietnam
[5] National Institute of Nutrition, Hanoi, Vietnam

* **Correspondence:** Email: tth6@huph.edu.vn, tranhai10.3@gmail.com.

Appendix 1

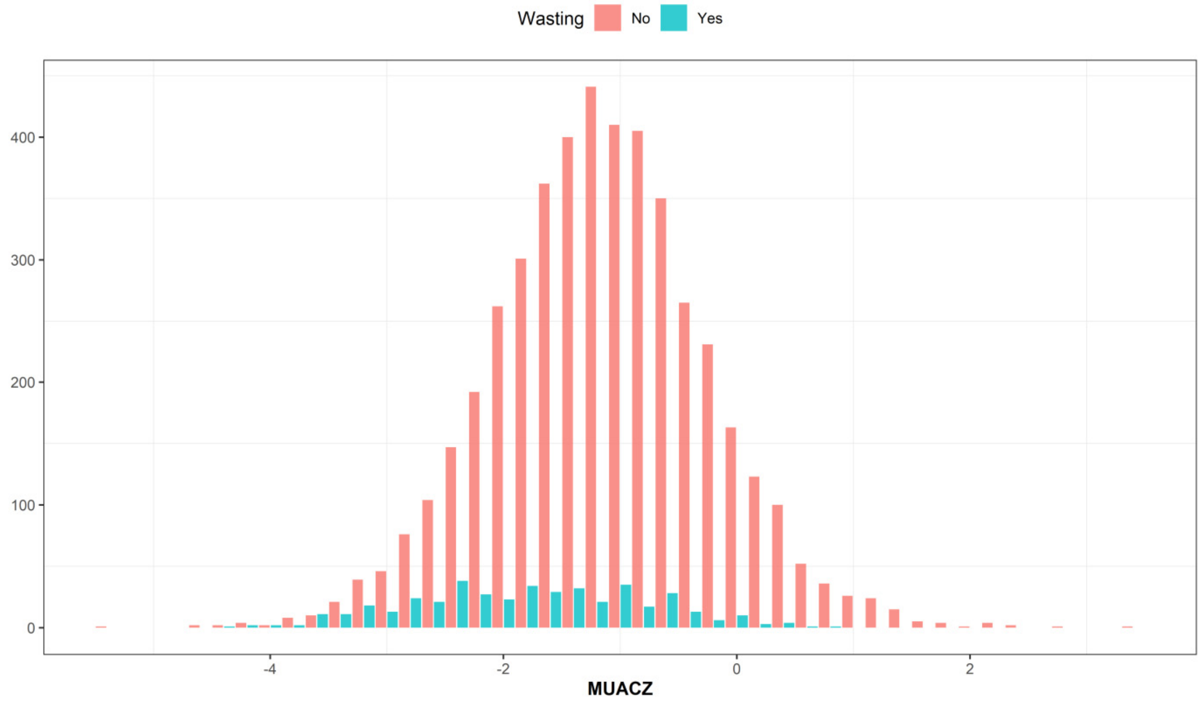

Figure 1. Histogram of the WHZ and MUACz distribution.

AIMS Press

© 2020 the Author(s), licensee AIMS Press. This is an open access article distributed under the terms of the Creative Commons Attribution License (http://creativecommons.org/licenses/by/4.0)
